# CCL24 Blockade with Nebokitug Modulates Inflammation and Fibrosis Signatures in Primary Sclerosing Cholangitis

**DOI:** 10.3390/cells15201853

**Published:** 2026-10-12

**Authors:** Revital Aricha, Tom Snir, John Lawler, Adi Mor

**Affiliations:** Chemomab Therapeutics Ltd., Tel Aviv 6158002, Israel

**Keywords:** CCL24, primary sclerosing cholangitis, inflammation, fibrosis

## Abstract

Background and Aims: Primary sclerosing cholangitis (PSC) is a chronic cholestatic liver disease characterized by progressive inflammation, fibrosis, and innate immune dysregulation, with no approved therapies. Nebokitug (CM-101), a humanized monoclonal antibody targeting CCL24, demonstrated anti-inflammatory, anti-fibrotic, and anti-cholestatic activity in a Phase 2 clinical trial in patients with PSC. We performed comprehensive serum proteomic analyses to characterize the mechanistic effects of the CCL24 blockade following treatment with nebokitug. Methods: Serum samples from study patients treated with nebokitug 10 mg/kg (n = 21), 20 mg/kg (n = 27), or placebo (n = 18) were analyzed using the Olink Explore 3072 proximity extension assay. Differential protein expression, pathway enrichment, and liver-specific proteome analyses were performed. Proteins corresponding to four PSC-specific gene expression programs (GEPs), identified previously by single-cell liver transcriptomics, were evaluated. An Elastic Net machine learning model was trained to classify treatment response defined by changes in Enhanced Liver Fibrosis (ELF) scores, liver stiffness measurements, and PRO-C3 test results. Results: Nebokitug induced dose-dependent modulation of proteins involved in chemotaxis, leukocyte recruitment, extracellular matrix remodeling, cytokine signaling, and collagen organization. Proteins representing PSC-specific GEPs related to fibrosis, extracellular matrix remodeling, macrophage activation, myofibroblast biology, and immune activation and were reduced following nebokitug treatment, particularly in patients with moderate-to-advanced disease treated with the 20 mg/kg dose. A machine-learning model accurately identified responders (mean AUROC = 0.92), with liver-specific proteins representing the dominant predictive signature. Consistent with this finding, analysis of liver-specific proteins demonstrated clear separation between responders and non-responders in the 20 mg/kg group (*p* = 0.0001), further linking treatment response to modulation of liver-specific biology. Conclusions: Comprehensive proteomic profiling demonstrates that a CCL24 blockade with nebokitug modulates key biological pathways driving PSC. These findings provide mechanistic support for nebokitug’s effect in PSC and further validate CCL24 as a promising therapeutic target in this indication.

## 1. Background

Primary sclerosing cholangitis (PSC) is a chronic immune-mediated cholestatic liver disease characterized by progressive inflammation and fibrosis of the intrahepatic and extrahepatic bile ducts, ultimately leading to cirrhosis, liver failure, and cholangiocarcinoma [1,2]. Despite substantial advances in understanding PSC biology, no pharmacological therapy has been approved to alter disease progression, and liver transplantation remains the only established treatment for patients with end-stage disease [1,2,3].

Accumulating evidence indicates that PSC is driven by persistent inflammation, innate immune activation, and progressive fibrogenesis. Hepatic macrophages have emerged as central regulators of disease progression through the production of inflammatory cytokines, chemokines, and profibrotic mediators that promote extracellular matrix (ECM) deposition and biliary fibrosis [4,5,6]. Consistent with these observations, several circulating macrophage-associated proteins have been associated with disease severity and adverse clinical outcomes in PSC [7], while macrophage stimulating protein-1 (MST1), a regulator of anti-inflammatory macrophage polarization, has been genetically linked to PSC susceptibility [8]. Recent single-cell and spatial transcriptomic analyses of human PSC liver tissue have revealed molecular networks involving immune activation, macrophage biology, extracellular matrix remodeling, and fibrogenic cell populations that contribute to PSC pathogenesis [9,10]. These molecular signatures are significantly enriched in PSC compared with healthy liver and other chronic liver diseases, suggesting that they represent biological pathways implicated in disease progression.

CCL24 (eotaxin-2) has emerged as an important upstream mediator linking inflammation and fibrosis. In addition to its established role in chemotaxis through CCR3 signaling, CCL24 promotes inflammatory cell recruitment and can directly modulate macrophage and fibroblast activity, contributing to downstream extracellular matrix deposition and fibrotic remodeling [11,12]. Experimental studies have demonstrated that inhibition of CCL24 attenuates inflammatory cell infiltration and tissue fibrosis, supporting CCL24 as a potential therapeutic target for chronic fibro-inflammatory diseases [11,12].

Nebokitug (CM-101) is a humanized monoclonal antibody that neutralizes CCL24. The Phase 2 SPRING clinical trial was a randomized, double-blind, placebo-controlled study evaluating nebokitug safety and activity in patients with PSC. The study demonstrated a favorable safety profile together with evidence of anti-inflammatory, anti-fibrotic, and anti-cholestatic activity after 15 weeks of treatment, including improvements in liver stiffness, Enhanced Liver Fibrosis (ELF) score and collagen turnover biomarkers [13]. These findings provided the first clinical evidence that CCL24 blockade may modulate key biological processes underlying PSC.

In the present study, we performed comprehensive serum proteomic profiling of patients enrolled in the Phase 2 SPRING trial using the Olink Explore platform. By integrating differential protein expression, pathway enrichment, protein-protein interaction networks, liver-specific protein signatures, PSC-specific gene expression programs, and machine-learning analyses, we sought to characterize the molecular effects of CCL24 blockade and identify biological pathways associated with response to nebokitug in patients with PSC.

## 2. Methods

### 2.1. Study Design and Population

Serum samples analyzed here were obtained from patients in the SPRING study (NCT04595825), a phase 2 randomized, double-blind, placebo-controlled, multiple-dose trial of nebokitug in large-duct PSC conducted at 33 centers across the United States, the United Kingdom, Germany, Spain, and Israel; the clinical results have been reported separately [13]. Eligible patients were aged 18–75 years with large-duct PSC of more than 24 weeks’ duration, defined by cholestatic serum liver tests with cholangiographic evidence of sclerosing cholangitis after exclusion of secondary causes, and a serum ALP of at least 1.5× the upper limit of normal; small-duct PSC, cirrhosis, a MELD score ≥16, competing liver etiologies, recent cholangitis or biliary instrumentation, and prior or planned liver transplantation were the key exclusions. Patients were randomized to intravenous nebokitug 10 mg/kg or 20 mg/kg, or matching placebo, every 3 weeks for 15 weeks, with dose cohorts enrolled sequentially and the 20 mg/kg cohort opening after a prespecified blinded interim safety review by the independent Data Safety Monitoring Committee.

### 2.2. Sample Collection

Serum was obtained from 66 SPRING patients (10 mg/kg n = 21, 20 mg/kg n = 27, placebo n = 18) at two timepoints: baseline (Visit 1) and Week 15 (Visit 6), corresponding to the start and end of the double-blind treatment period. A separate control cohort of 30 healthy individuals, matched to the patient population for age and sex (10 females, age 41.8 ± 4.1 years (mean ± SD), range 37–50; 20 males, age 40.1 ± 4.6 years, range 35–50; overall age 40.7 ± 4.5 years, range 35–50), was profiled at a single timepoint; these samples were obtained from a commercial biorepository (Precision Biospecimen Solutions, Inc., Bethesda, MD, USA). The study was conducted in accordance with the Declaration of Helsinki and approved by the relevant Institutional Review Boards, with written consent obtained from every patient before sample collection. Alongside the serum, demographic and clinical variables were recorded for each patient, including age, sex, serum biochemistry values, complete blood counts, and any evidence of or history of malignancy or disease-related symptoms.

### 2.3. Proteomic Quantification

Serum protein levels were measured using the Olink Explore 3072 platform (Olink Proteomics, Uppsala, Sweden), a proximity extension assay spanning eight panels (Cardiometabolic I/II, Inflammation I/II, Neurology I/II, Oncology I/II) and 2943 assays. Protein abundance is reported as Normalized Protein eXpression (NPX), an arbitrary unit on a log2 scale.

### 2.4. Data Processing

Proteomic data were processed in R (version 4.6.1; R Foundation for Statistical Computing, Vienna, Austria) using the Olink Analyze package (version 5.0.2). Of the 2943 assays, 84 were flagged as excluded by Olink’s quality control and returned no NPX values; these were removed, leaving 2859 assays. Six proteins (CXCL8, IDO1, IL6, LMOD1, SCRIB and TNF) are measured on four of the eight panels as inter-panel bridging controls; for these, a single assay was retained per protein. This yielded 2841 unique proteins entering analysis. Finally, the pharmaceutical target, CCL24, was excluded from the analysis. NPX values are reported on a log2 scale, and differences in NPX therefore correspond to log2 fold changes. For each patient in the active and placebo arms, change from baseline (ΔNPX) was calculated per protein as the Week 15 (Visit 6) value minus the baseline (Visit 1) value. Healthy control samples were obtained at a single timepoint and contributed baseline NPX values only.

### 2.5. Differential Expression Analysis

Treatment effects were estimated per protein using linear mixed-effects models of NPX. Fixed effects included: treatment arm (nebokitug 10 and 20 mg/kg pooled vs. placebo), visit, the treatment-by-visit interaction, and baseline liver stiffness stratum (≤8.7 vs. >8.7 kPa) as a stratification covariate; a patient-level random intercept accounted for repeated measurements. Dose arms were pooled to preserve statistical power. Models were fitted independently for each of the 2840 proteins, and the treatment-by-visit interaction term was used to test the treatment effect. Observations with missing NPX were excluded listwise by the fitting procedure.

*p*-values were adjusted across all proteins by the Benjamini–Hochberg method to control the false discovery rate (FDR). Proteins with FDR < 0.05 were considered statistically significant; proteins reaching unadjusted *p* < 0.01 without surviving FDR correction are reported as exploratory and were not interpreted as confirmatory. Effect sizes are reported as the placebo-corrected log2 fold change at Week 15, calculated as the between-arm difference in mean ΔNPX; because NPX is a log2-scale measure, this difference is itself a log2 fold change. Effect sizes and *p*-values therefore derive from a descriptive contrast and the mixed model respectively.

### 2.6. Pathway Enrichment Analysis

To identify biological processes associated with the treatment effect, all measured proteins were ranked by their placebo-corrected log2 fold change at Week 15, defined as the mean change from baseline in the pooled active arms minus that in placebo. No significance threshold was applied to the protein-level results before enrichment, so the full ranked distribution contributed to the analysis. Gene set enrichment analysis was performed with STRING (version 12.0), testing for enrichment against Gene Ontology Biological Process and Molecular Function annotations, with the analysis restricted to gene sets containing 20 to 500 proteins. Terms with FDR < 0.05 were considered statistically significant.

### 2.7. Protein–Protein Interaction Network Analysis

Proteins reduced at Week 15 in the pooled active arms relative to placebo at unadjusted *p* < 0.01 (n = 121) were used as seed nodes to construct a protein–protein interaction network in NetworkAnalyst 3.0, drawing on the STRING interactome. A minimum-order network was used to restrict expansion to the connecting nodes essential to link the seed proteins, limiting network size and avoiding hub-driven inflation. Functional enrichment of the resulting network was then performed against Gene Ontology Biological Process and Molecular Function annotations, with terms at FDR < 0.05 considered significant.

### 2.8. Extracellular Matrix Remodeling Analysis

Core matrisome proteins were defined by the NABA_CORE_MATRISOME gene set retrieved from the Molecular Signatures Database (MSigDB) collection C2 using the msigdbr package (version 26.1.0) and intersected with the measured proteome by gene symbol.

Two orthogonal contrasts were computed for each protein. The disease effect was estimated by fitting a linear model of absolute NPX on disease status, comparing baseline values from patients with PSC against healthy controls; *p*-values were adjusted across all proteins by the Benjamini–Hochberg method. The treatment effect was the placebo-corrected log2 fold change at Week 15 described previously, with its associated unadjusted *p*-value from the mixed model. Proteins were classified as showing reversal of the disease signature when they were significantly elevated in PSC relative to healthy controls (FDR < 0.05) and reduced by treatment, at nominal *p* < 0.05 or, as a secondary tier, *p* < 0.10. Nominal thresholds were used for the treatment axis because this analysis was descriptive and hypothesis-generating rather than confirmatory.

To test whether the core matrisome was preferentially modulated relative to the remainder of the measured proteome, placebo-corrected log2 fold changes were compared between core matrisome proteins and all other proteins using the Wilcoxon rank-sum test.

### 2.9. Gene Expression Program Analysis

Four PSC-associated gene expression programs (GEPs)—GEP16 (fibroblast/myofibroblast, matrix-producing), GEP18 (monocyte/macrophage, fibrotic), GEP24 (fibroblast/myofibroblast, Wnt antagonism) and GEP32 (immune activation, CXCL10^+^ signaling) were taken from Herb et al. [10], who defined them by spatially resolved single-cell analysis of PSC liver explants using consensus non-negative matrix factorization [14]. Each program was represented in serum by its constituent proteins present on the Olink panel: 4, 5, 4 and 4 proteins for GEP16, GEP18, GEP24 and GEP32 respectively (17 in total; all listed program members were measured).

Two analyses were performed: First, at baseline, each patient was assigned a single score per program, calculated as the mean NPX across that program’s proteins, and scores were compared between mild disease (≤8.7 kPa, n = 30) and moderate-to-advanced disease (>8.7 kPa, n = 36) by Welch’s two-sample *t*-test; *p*-values were adjusted across the four programs by the Benjamini–Hochberg method. Second, within the moderate-to-advanced disease stratum, the mean change from baseline to Week 15 (ΔNPX ± SE) was displayed for each constituent protein by treatment arm (placebo n = 8, 10 mg/kg n = 14, 20 mg/kg n = 14).

### 2.10. Machine Learning Model of Composite Response

A composite response improvement outcome was defined for actively treated patients (10 and 20 mg/kg, pooled) as a change from baseline in ELF score < 0.19 [15], together with any reduction from baseline in liver stiffness measurement by transient elastography and in PRO-C3 result. Predictors were baseline-to-Week 15 changes (ΔNPX) in all measured proteins, excluding CCL24 as the pharmacodynamic target. Proteins with missing values in more than 50% of patients were excluded; remaining missing values were imputed using k-nearest neighbors, and all features were centered and scaled within each cross-validation training fold.

A penalized logistic regression model (Elastic Net) was fitted using glmnet (version 5.0) through the caret framework (version 7.0.1). The mixing parameter α was tuned from 0 to 0.5 in increments of 0.1, and the penalty parameter λ was tuned over 50 values spanning 10^−4^ to 10, selected to maximize cross-validated AUROC. Class imbalance was addressed by upsampling the minority class within each training fold. Model fitting was repeated across 500 iterations of repeated five-fold cross-validation; performance is reported as the mean and standard deviation of cross-validated AUROC at the selected hyperparameters, together with sensitivity, specificity and balanced accuracy.

Feature importance was aggregated across the 500 iterations by recording proteins with non-zero coefficients of each fitted model. Proteins selected in at least 50% of iterations constituted the consensus signature, for which selection frequency and median coefficient are reported. Separation according to response status was displayed by principal component analysis of ΔNPX values restricted to the consensus proteins, and by a heatmap of row-scaled ΔNPX values.

Model validity was assessed by permutation testing, in which outcome labels were randomly permuted across 100 iterations, and the complete modeling procedure was repeated; the empirical *p*-value was calculated as the proportion of permuted AUROCs greater than or equal to the observed AUROC.

### 2.11. Liver-Specific Protein Analysis

To assess liver-specific proteomic changes, proteins were restricted to a liver-enriched set defined by the MSP liver atlas [16] and mapped to the Olink panel by UniProt identifier, yielding 212 measured proteins. Principal component analysis was performed on change from baseline to Week 15 (ΔNPX) in patients receiving nebokitug 20 mg/kg, classified by composite response status. Proteins with valid values in fewer than 70% of patients, and those with zero variance, were excluded; patients with any remaining missing value were removed listwise, giving a final matrix of 205 proteins in 27 patients (10 responders, 17 non-responders).

### 2.12. Software and Statistical Analysis

All analyses were performed in R version 4.6.1. Linear mixed-effects models were fitted with the Olink Analyze package (version 5.0.2), which implements model fitting via lme4 (version 2.0.6) and lmerTest (version 3.2.1). Principal component analysis was performed with FactoMineR (version 2.16) and visualized with factoextra (version 2.2.0). Matrisome annotation was obtained through msigdbr (version 26.1.0). Penalized logistic regression models were fitted with glmnet (version 5.0) within the caret framework (version 7.0.1), and classification performance was assessed with pROC (version 1.19.0.1). Data manipulation used the tidyverse collection (version 2.0.0), and figures were produced with ggplot2 (version 4.0.3), with ggrepel (version 0.9.8), ggsignif (version 0.6.4) and patchwork (version 1.3.2). Gene set enrichment and protein–protein interaction network analyses were performed using the STRING and NetworkAnalyst web platforms, respectively.

## 3. Results

### 3.1. Treatment Affects Proteomic Profile of Patients with PSC

Among the 2840 proteins analyzed, 12 were differentially modulated by nebokitug relative to placebo at an FDR < 0.05 (Figure 1). CXCL11, CAPN3, MB, FAM171A2, HGFAC, PSAP, MEP1B, SHBG, NRN1, ATP1B1 and VNN1 were decreased, whereas MST1 was increased. An additional 116 proteins reached nominal significance (unadjusted *p* < 0.01) but did not meet the FDR threshold and were therefore considered exploratory signals.

### 3.2. Nebokitug Reduces Extracellular Matrix and Immune Signaling Programs

Proteins reduced by nebokitug relative to placebo were enriched for extracellular matrix and tissue-remodeling processes. Enriched GO Molecular Function terms included structural constituent of muscle, immune receptor activity, growth factor binding, glycosaminoglycan binding, transmembrane receptor protein kinase activity and cytokine receptor activity (Figure 2A, upper panel). GO Biological Process terms were similarly enriched and included, comprising cell–cell adhesion via plasma-membrane adhesion molecules, muscle contraction, muscle system process, blood vessel morphogenesis, extracellular matrix organization and wound healing (Figure 2A, lower panel). A protein–protein interaction network analysis seeded by the down-regulated proteins identified similar biological programs, with growth factor and glycosaminoglycan binding, cytokine receptor binding and activity, and wound healing, cell activation and cell migration among the enriched terms (Figure 2B). Together, these results indicate coordinated suppression of matrix organization, growth factor activity, and immune receptor signaling following CCL24 blockade.

### 3.3. MST1 Is Reduced in PSC and Increases Following Treatment with Nebokitug

MST1 is a negative regulator of macrophage-driven inflammation with established genetic linkage to PSC susceptibility [8] and has been implicated in the regulation of inflammatory responses, with studies of the MST1/MSP–RON pathway demonstrating its involvement in modulating inflammation and tissue injury across multiple organs, including the gut and liver [17]. MST1 was reduced in patients with PSC relative to healthy controls (*p* = 0.0065; Figure 3). Nebokitug treatment increased MST1 in a dose-dependent manner: mean ΔNPX at Week 15 was 0.27 in the placebo arm, 0.69 at 10 mg/kg and 1.06 at 20 mg/kg (*p* < 0.0001 for 20 mg/kg vs placebo). Levels in both active arms shifted upward toward those observed in healthy controls. Increased MST1 following CCL24 blockade is consistent with modulation of the macrophage-associated inflammatory biology implicated in PSC disease progression.

### 3.4. Proteins Representing PSC-Specific GEPs Are Elevated in Moderate-to-Advanced Disease and Reduced by Nebokitug

Recent single-cell analyses of human PSC liver tissue defined four PSC-specific gene expression programs (GEPs) spanning fibrosis/myofibroblast matrix production, myofibroblast Wnt antagonism, fibrotic macrophage activity, and CXCL10^+^ immune activation [6]. To assess whether nebokitug affects these programs, the corresponding serum proteins were evaluated by baseline disease severity and by treatment.

At baseline, the fibrosis/myofibroblast matrix-producing (*p* = 0.0016) and CXCL10^+^ immune-activation (*p* = 0.041) programs were elevated in patients with moderate-to-advanced disease (liver stiffness > 8.7 kPa) relative to those with mild disease (Figure 4A); the fibrotic macrophage and Wnt-antagonism programs did not differ by severity. Among patients with moderate-to-advanced disease, proteins across all four GEPs showed dose-dependent reductions in the active arms relative to placebo, with the largest effects at 20 mg/kg (Figure 4B). These findings further support an effect of CCL24 blockade on serum proteins associated with the core fibrotic and immune-activation programs that characterize PSC liver pathology.

### 3.5. Treatment with Nebokitug Shifts Circulating Core Matrisome Proteins Towards Levels Seen in Healthy Individuals

Given that progressive ECM remodeling and fibrosis are central features of PSC pathogenesis, we specifically examined circulating core matrisome proteins to determine whether CCL24 blockade modulates this disease-relevant biological compartment. Each protein was positioned on two axes: a disease axis, comparing baseline levels in patients with PSC with those in healthy controls, and a treatment axis, the placebo-corrected log_2_ fold change at Week 15. Proteins elevated in disease and reduced by treatment were located in the lower-right quadrant (Figure 5A).

Of 114 core matrisome proteins measured, 23 were significantly elevated in PSC relative to healthy controls (FDR < 0.05) and reduced following treatment at nominal *p* < 0.1, of which 15 reached nominal *p* < 0.05. These included CTHRC1, CCN1, IGFBP1, COL2A1 and IGFBP7, proteins associated with hepatic fibrogenesis, as well as PRG2, a finding consistent with decreased eosinophil activation downstream of CCL24 blockade [18]. No protein reached FDR-adjusted significance on the treatment axis; per-protein treatment effects are therefore reported as exploratory.

A general downward shift was observed across the proteome, with 71.1% of the 2725 non-matrisome proteins showing a negative placebo-corrected fold change (median −0.111). Core matrisome proteins showed a greater downward shift, with 89.5% reduced and a median of −0.152 (Figure 5B). The distribution of placebo-corrected fold changes differed between core matrisome proteins and the remainder of the measured proteome (Wilcoxon rank-sum, *p* = 3.9 × 10^−4^), suggesting that the reduction in matrisome proteins was not fully explained by the global shift alone.

### 3.6. A Machine-Learning Model Identifies a Protein Signature Associated with Composite Clinical Response to Nebokitug

To determine whether treatment-associated proteomic changes can track clinical benefit, a machine-learning model was trained to distinguish patients according to a composite improvement outcome, defined by concurrent reductions in ELF score, liver stiffness, and PRO-C3. Using baseline-to-Week 15 changes across all measured proteins, a penalized logistic regression (Elastic Net) model distinguished patients who met the composite improvement criterion from those who did not, achieving a mean AUROC of 0.92 across repeated cross-validation. Permutation testing confirmed model validity, exceeding null models trained on randomized labels (mean null AUROC 0.59; *p* < 0.05).

Feature importance aggregated over 500 iterations of repeated 5-fold cross-validation yielded a stable signature of 14 proteins consistently selected across iterations (Figure 6A). Principal component analysis (PCA) using this 14-protein signature separated patients achieving composite improvement from those who did not (Figure 6B), indicating that a compact, liver-enriched protein set captures the treatment response. Per-patient profiles of the signature proteins showed a consistent difference between responders (n = 14) and non-responders (n = 34) (Figure 6C).

### 3.7. A Liver-Specific Protein Signature Separates Treatment Responders from Non-Responders

Building on these findings, we examined whether changes in liver-enriched proteins differed between treatment responders and non-responders. Liver-enriched proteins were identified from a published single-cell liver atlas [16] and mapped to the Olink panel using UniProt identifiers; 212 of these proteins were measured, of which 205 met the completeness and variance criteria. Principal component analysis of change from baseline to Week 15 in patients receiving nebokitug 20 mg/kg (n = 27), classified by composite response (concurrent improvement in ELF, liver stiffness, and PRO-C3), separated responders (n = 10) from non-responders (n = 17) along the second principal component (16.2% of variance; *p* = 0.0001) (Figure 7). Separation was weaker in the 10 mg/kg arm and in the pooled active arms, consistent with a dose-dependent effect. These findings suggest that response to CCL24 blockade is associated with coordinated changes in liver-enriched circulating proteins.

## 4. Discussion

In this study, we performed an integrated proteomic analysis of serum samples from patients enrolled in the Phase 2 SPRING trial to characterize the molecular effects of CCL24 blockade in PSC. By combining high-dimensional proteomics with pathway enrichment, liver-specific protein signatures, PSC-associated transcriptomic programs, and machine-learning approaches, we demonstrate that treatment with nebokitug is associated with coordinated modulation of biological pathways implicated in PSC pathogenesis. Rather than affecting isolated biomarkers, CCL24 inhibition was associated with broad changes across interconnected inflammatory and fibrogenic networks, providing mechanistic support for the clinical activity previously observed in the SPRING trial [13].

One of the principal findings of this study is the dose-dependent reduction in proteins involved in immune cell recruitment, extracellular matrix remodeling, cytokine signaling, and collagen organization. These biological processes have consistently emerged as hallmarks of PSC disease progression in both experimental models and human liver tissue studies [4,5,6,7,8,9,10]. Recent single-cell and spatial transcriptomic analyses have highlighted the importance of coordinated interactions between immune cells, fibrogenic stromal populations, and cholangiocytes in sustaining chronic biliary inflammation and fibrosis [9,10]. Our findings demonstrate that pharmacological inhibition of CCL24 is associated with modulation of many of these same molecular pathways in patients, suggesting that the circulating proteome reflects biologically meaningful changes related to the diseased liver [9,10,19].

Importantly, the proteins modulated by nebokitug substantially overlapped with molecular programs previously identified in PSC liver tissue [9,10]. Proteins representing pathways associated with immune activation, extracellular matrix remodeling, macrophage biology, and fibrogenic stromal responses were elevated in patients with moderate-to-advanced disease and were preferentially reduced following treatment, particularly in the 20 mg/kg treatment group. The observation that these disease-associated molecular signatures are reversible following CCL24 blockade provides translational evidence linking target engagement to modulation of biological pathways implicated in PSC pathogenesis.

Gene set enrichment and protein interaction network analyses consistently demonstrated downregulation of pathways involved in leukocyte migration, chemokine activity, cell–cell adhesion, extracellular matrix organization, growth factor signaling, and collagen binding. These findings are consistent with the known biology of CCL24, which promotes recruitment and activation of inflammatory leukocytes and fibroblasts through CCR3 signaling and contributes to extracellular matrix deposition in multiple fibrotic diseases [11,12]. Collectively, these data suggest that CCL24 functions as an upstream regulator of multiple biological processes rather than a single inflammatory pathway, potentially explaining the broad molecular changes observed following nebokitug treatment.

A focused analysis of the circulating core matrisome further supports preferential modulation of fibrosis-related biology by CCL24 blockade. Consistent with the central role of extracellular matrix remodeling in PSC, circulating core matrisome proteins exhibited a preferential shift toward the healthy molecular profile following nebokitug treatment. This effect was significantly greater than the overall proteomic response, suggesting selective modulation of fibrosis-associated biology rather than a generalized reduction in circulating proteins.

An additional aspect of this study is the integration of liver-specific proteomic signatures with machine-learning analyses. Using an Elastic Net approach, we identified a reproducible protein signature that distinguished patients demonstrating a composite treatment response, defined by simultaneous improvements in liver stiffness measurements, ELF scores, and PRO-C3 results. The high cross-validated performance of the model (mean AUROC = 0.92), together with the results of permutation testing, suggests that treatment response is characterized by coordinated biological changes rather than stochastic variation. Notably, liver-specific proteins represented the dominant contributors to the predictive signature, supporting the concept that circulating proteomic changes may reflect treatment-associated changes in hepatic biology rather than nonspecific systemic effects [16]. Consistent with this finding, the significant separation of responders and non-responders based on liver-enriched proteins defined using a single-cell liver atlas provides additional evidence linking treatment response to coordinated modulation of liver-specific biology.

The concordance between proteomic findings and clinical outcome measures further strengthens the biological relevance of our observations. The composite response incorporated complementary measures of fibrosis, including liver stiffness measurements, ELF scores, and PRO-C3 results, each reflecting distinct aspects of extracellular matrix remodeling [7,20,21,22,23]. The association between coordinated proteomic changes and improvements across these independent clinical biomarkers suggests that the observed molecular effects are associated with treatment-related improvements in liver disease biology.

Several limitations should be acknowledged. The analyses were performed on serum collected over a relatively short 15-week treatment period and involved a modest number of patients, reflecting the size of the Phase 2 study. While the consistency across multiple analytical approaches supports the robustness of the findings, external validation in independent PSC cohorts and larger clinical trials will be important to confirm these molecular signatures. Furthermore, although serum proteomics provides a non-invasive window into disease biology, direct confirmation of these molecular changes within liver tissue following treatment was beyond the scope of the current study.

In conclusion, comprehensive proteomic profiling demonstrates that CCL24 blockade with nebokitug induces coordinated modulation of molecular pathways central to PSC pathogenesis, including immune activation, extracellular matrix remodeling, and liver-specific biological processes. These findings extend clinical observations from the SPRING clinical trial by providing mechanistic evidence that inhibition of CCL24 modulates disease-associated molecular networks beyond individual biomarkers.

## Figures and Tables

**Figure 1:**
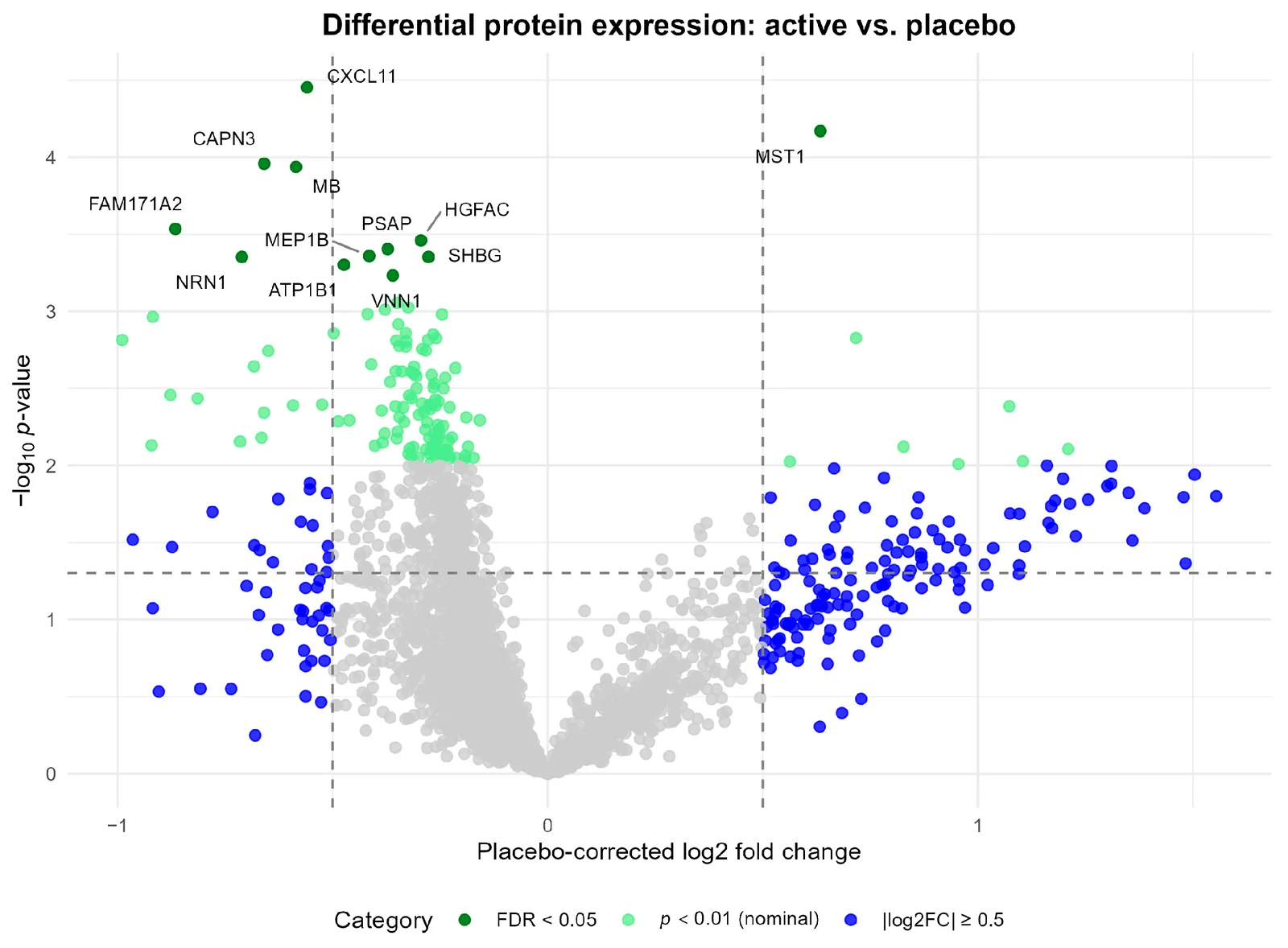
Differential serum protein expression in nebokitug-treated patients relative to placebo at Week 15. Placebo-corrected log_2_ fold change is plotted against the unadjusted −log_10_ *p*-value from the per-protein linear mixed-effects model (treatment-by-visit interaction). Dark green, FDR < 0.05; light green, nominal *p* < 0.01; blue, |log_2_ fold change| ≥ 0.5; grey, all other proteins. Dashed lines mark log_2_ fold changes of ±0.5 and *p* = 0.05. The 12 proteins meeting FDR < 0.05 are labeled.

**Figure 2:**
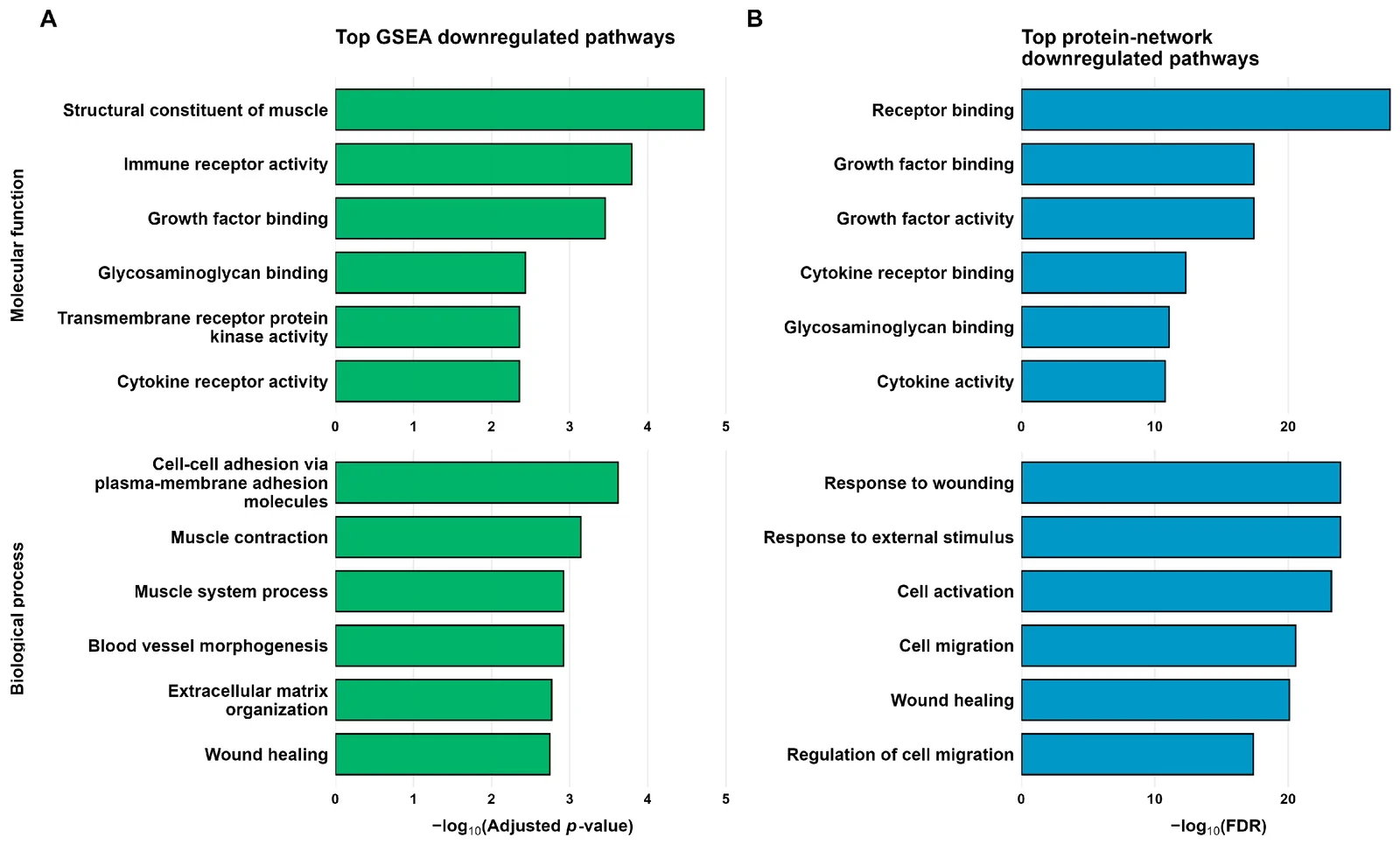
Biological pathways associated with proteins reduced by nebokitug relative to placebo at Week 15. (**A**) Gene set enrichment analysis (STRING) of all measured proteins ranked by placebo-corrected log_2_ fold change, showing the six most significantly enriched Gene Ontology Molecular Function (upper) and Biological Process (lower) terms among down-regulated proteins. (**B**) Functional enrichment of a protein–protein interaction network (NetworkAnalyst) seeded by proteins reduced at unadjusted *p* < 0.01, showing the six most significant Molecular Function (upper) and Biological Process (lower) terms. Bars are ordered by significance.

**Figure 3:**
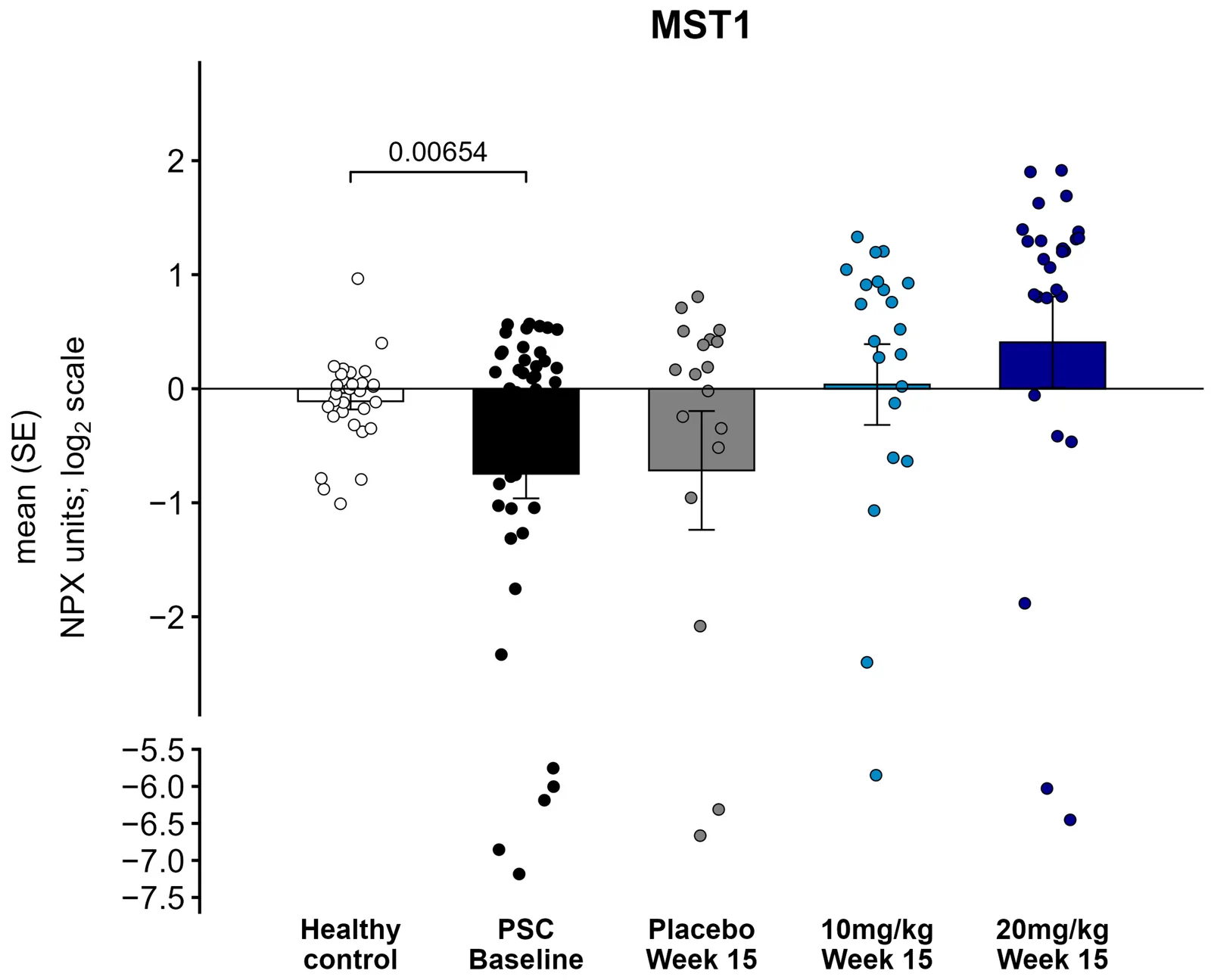
Serum MST1 levels in healthy controls and in patients with PSC before and after treatment. Absolute NPX values (log_2_ scale) are shown for healthy controls, patients with PSC at baseline, and the placebo, 10 mg/kg and 20 mg/kg arms at Week 15. Bars show the group mean with standard error; individual samples are overlaid as points. The bracket indicates the Welch t-test *p*-value for healthy controls versus PSC baseline.

**Figure 4:**
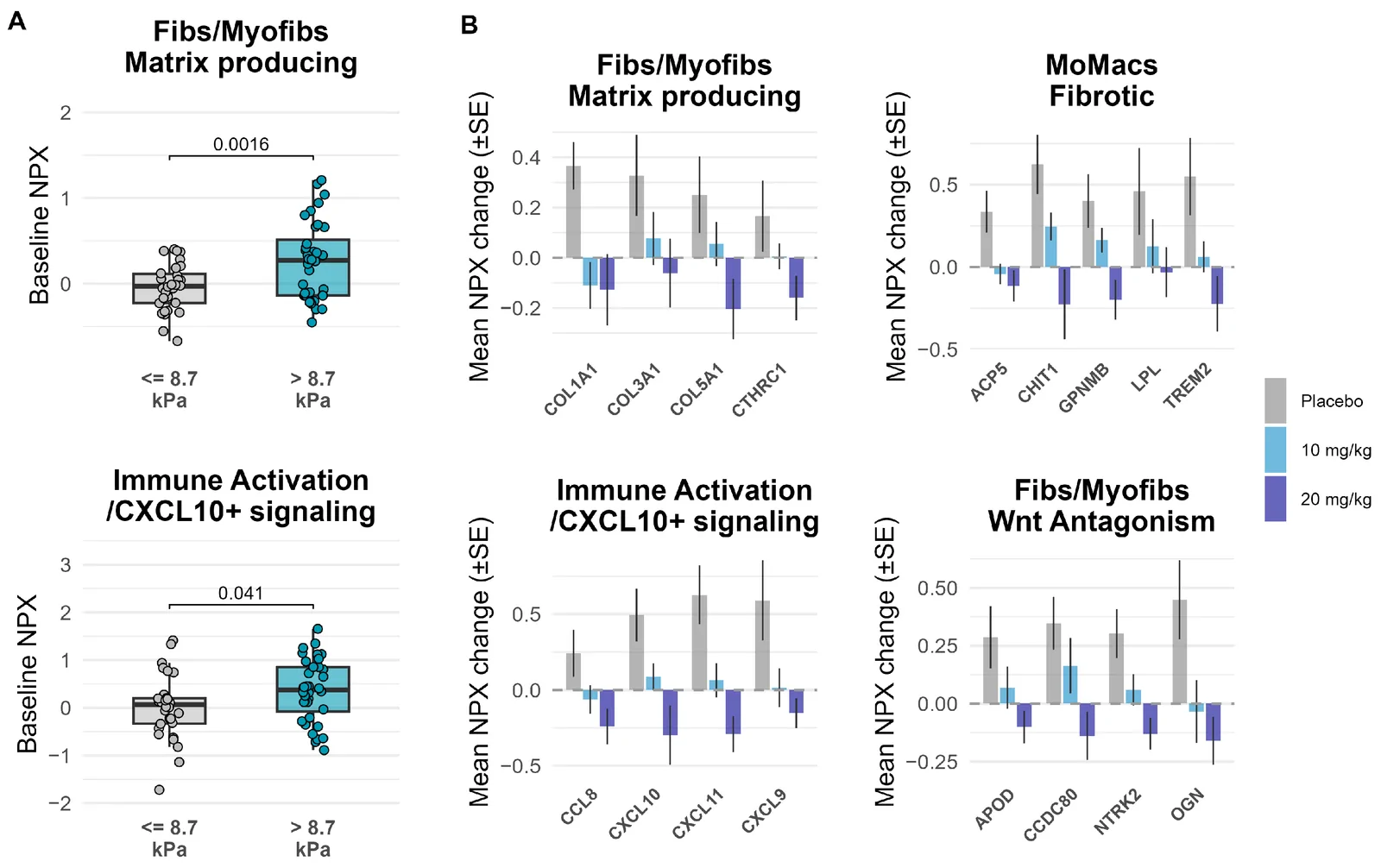
Proteins representing PSC-specific gene expression programs (GEPs) in relation to disease severity and to nebokitug treatment. (**A**) Baseline NPX averaged across the proteins of each GEP per patient, compared between patients with mild (≤8.7 kPa) and moderate-to-advanced (>8.7 kPa) baseline liver stiffness; boxes show the median and interquartile range with individual patients overlaid, and brackets give Welch t-test *p*-values. Only the two programs that differed by severity are shown. (**B**) Mean change in NPX from baseline to Week 15 (±SE) for the individual proteins of each of the four GEPs, by treatment arm, restricted to patients with baseline liver stiffness > 8.7 kPa.

**Figure 5:**
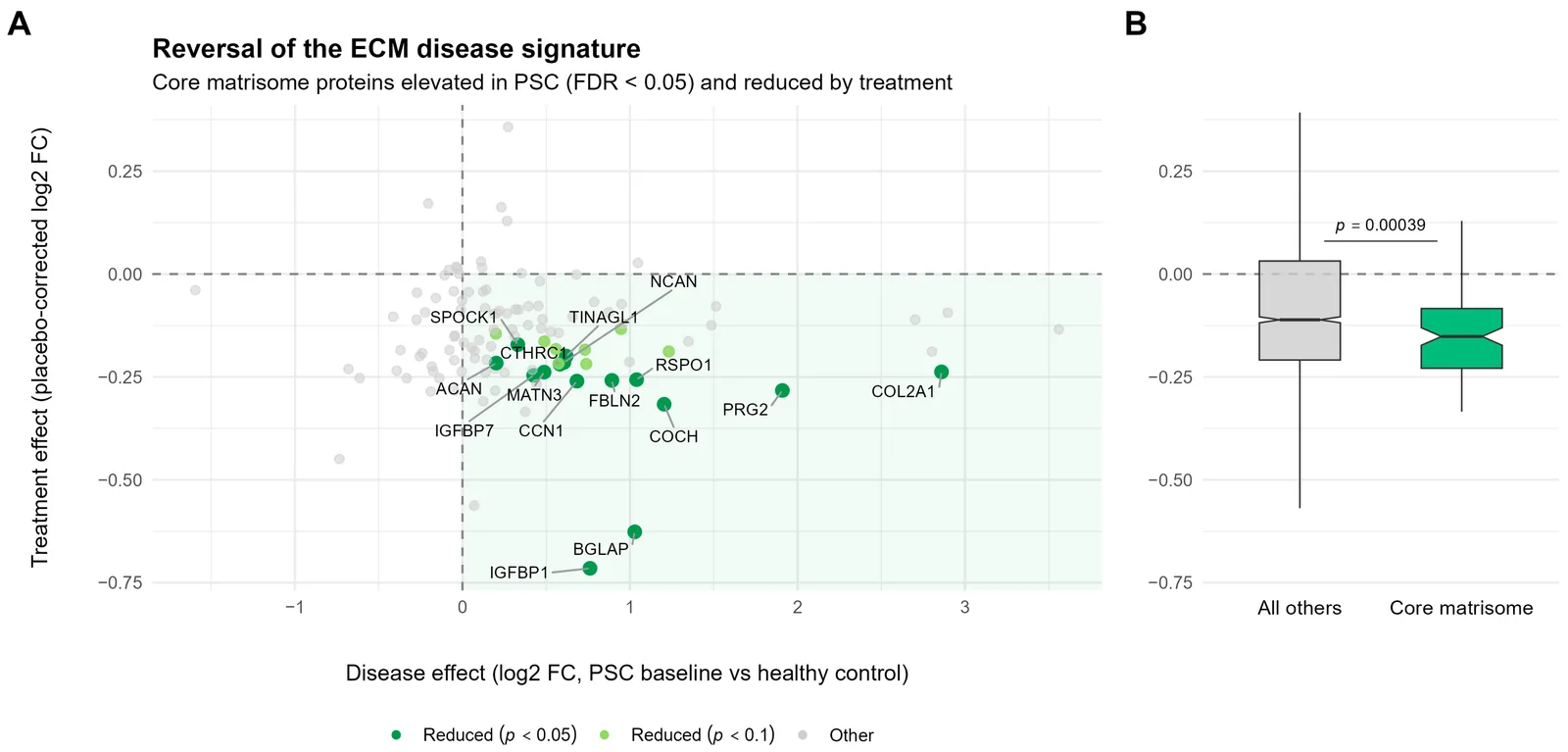
Core matrisome proteins are elevated in PSC and reduced by nebokitug. (**A**) Each core matrisome protein positioned by its disease effect (log_2_ fold change, PSC baseline versus healthy controls; x-axis) and its treatment effect (placebo-corrected log_2_ fold change at Week 15; y-axis). The shaded lower-right quadrant contains proteins elevated in disease and reduced by treatment. Dark green, proteins elevated in PSC at FDR < 0.05 and reduced at nominal *p* < 0.05; light green, the same disease criterion with reduction at nominal *p* < 0.10; grey, all other core matrisome proteins. Only the dark green proteins are labeled. (**B**) Distribution of placebo-corrected log_2_ fold changes for core matrisome proteins versus the remainder of the measured proteome; boxes show the median, interquartile range and notched 95% confidence interval of the median, with the Wilcoxon rank-sum *p*-value.

**Figure 6:**
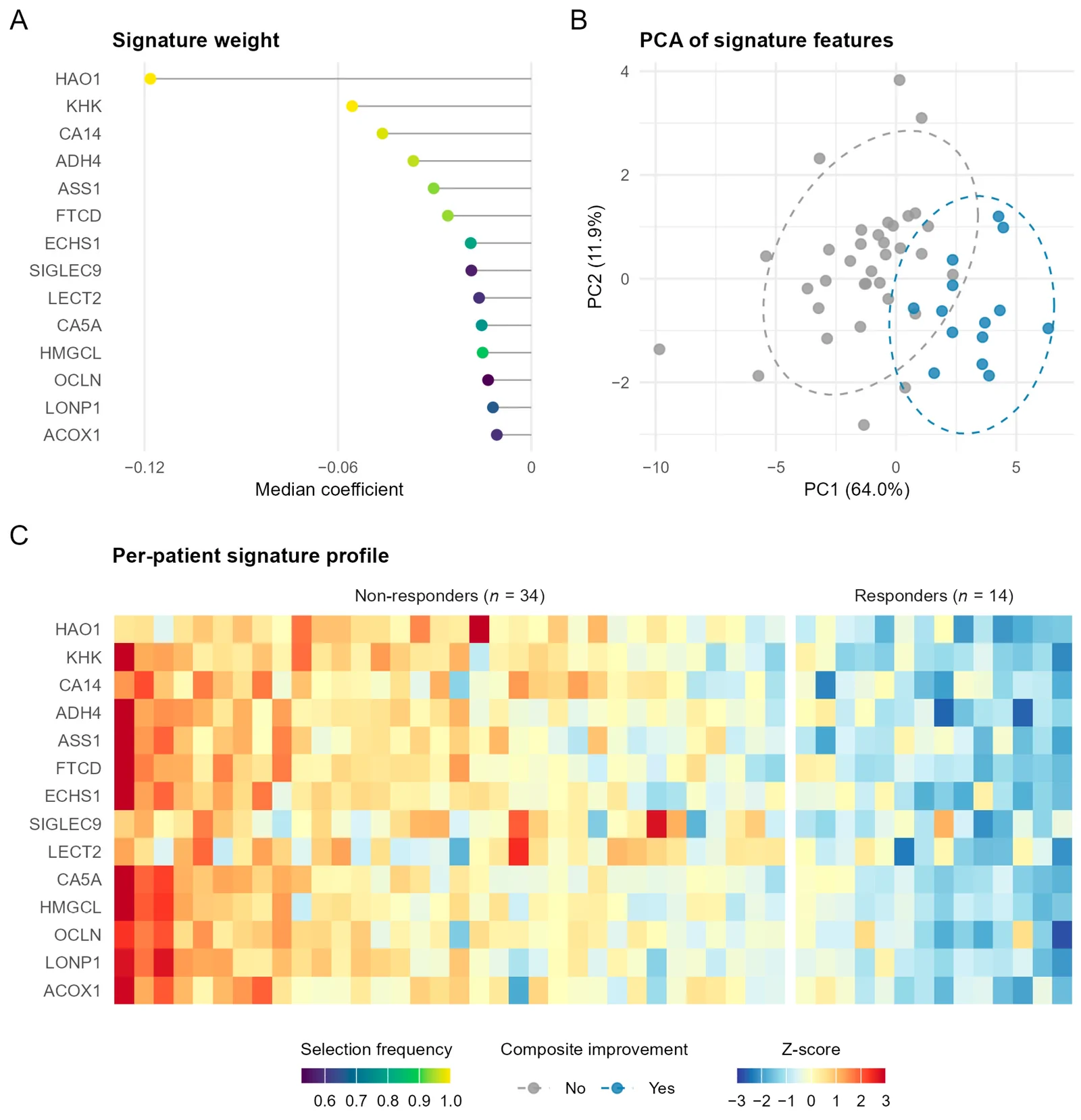
Elastic Net classification of composite treatment response in actively treated patients. (**A**) The 14-protein stable signature, ordered by absolute median coefficient across 500 iterations of repeated five-fold cross-validation; point color indicates the frequency with which each protein was selected across iterations. (**B**) Principal component analysis of baseline-to-Week 15 changes in the 14 signature proteins, with patients colored by composite improvement status; dashed lines are 95% confidence ellipses. (**C**) Per-patient profile of the same 14 proteins, shown as z-scored changes from baseline, with patients grouped by composite response status.

**Figure 7:**
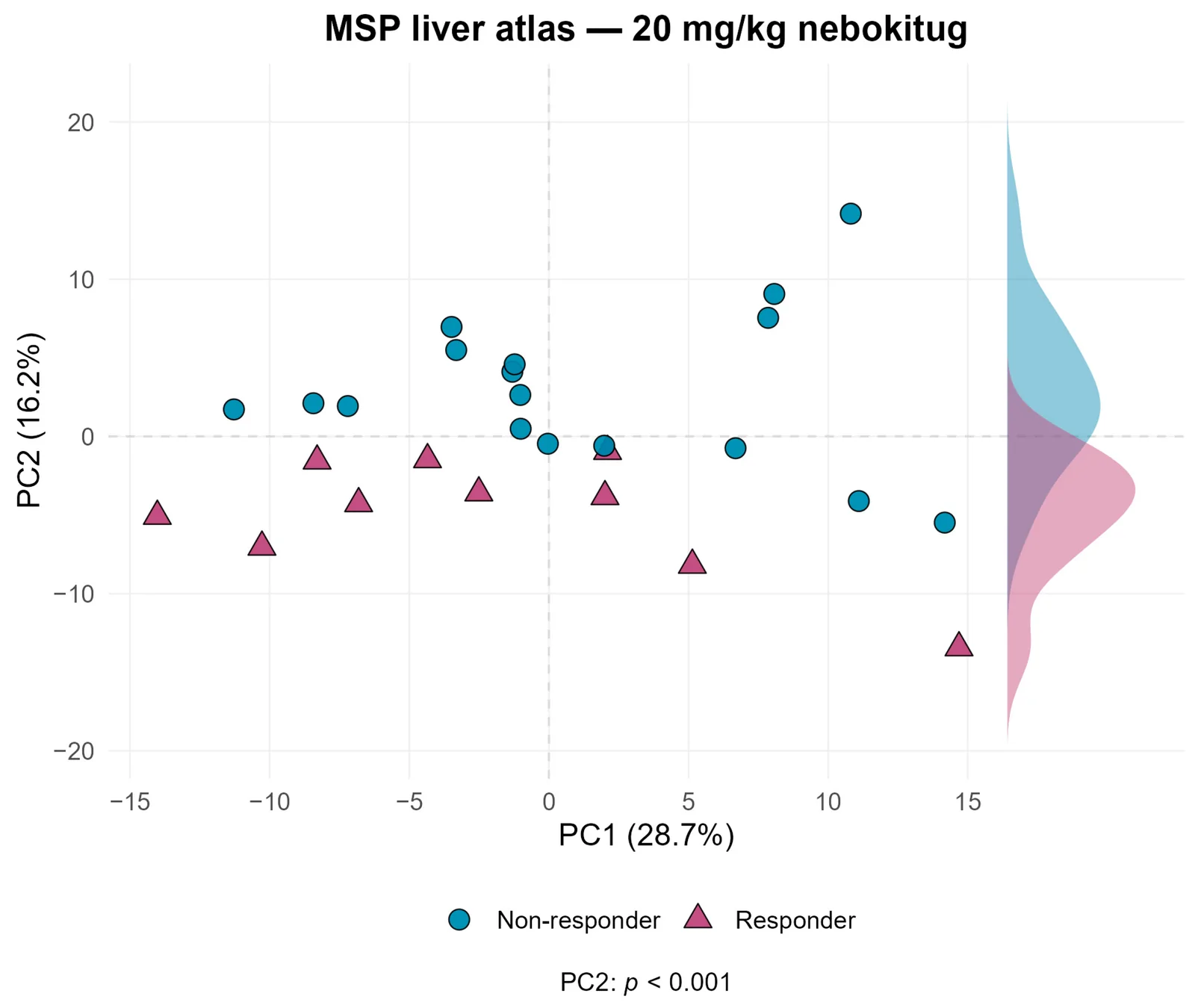
Liver-enriched proteomic changes separate responders from non-responders in the 20 mg/kg arm. Principal component analysis of baseline-to-Week 15 changes in NPX across 205 liver-enriched proteins, in patients receiving nebokitug 20 mg/kg, classified by composite response (concurrent improvement in ELF score, liver stiffness and PRO-C3). Each point is one patient; the proportion of variance explained is shown on each axis. Marginal density curves on the right show the distribution of PC2 scores by response group, and the Welch *t*-test *p*-value for the difference in PC2 scores is given below the plot.

## Data Availability

Summary results of the SPRING trial (NCT04595825) have been reported previously [13]. The individual-level clinical and proteomic data generated and analyzed in the present study derive from an ongoing clinical development program and are subject to confidentiality restrictions; they are therefore not publicly available. Requests for access to the data supporting the reported results may be directed to the corresponding author and will be considered on a case-by-case basis, subject to applicable confidentiality obligations and to the protection of patient privacy.

## Abbreviations

The following abbreviations are used in this manuscript:

AUROC	Area under the receiver operating characteristic curve
CCL24	C-C motif chemokine ligand 24
CCR3	C-C chemokine receptor type 3
ECM	Extracellular matrix
ELF	Enhanced liver fibrosis
FDR	False discovery rate
GEP	Gene expression program
GO	Gene Ontology
MSigDB	Molecular Signatures Database
MST1	Macrophage stimulating protein 1
NPX	Normalized protein expression
PCA	Principal component analysis
PRO-C3	N-terminal propeptide of type III collagen
PSC	Primary sclerosing cholangitis
